# Low level expression of the Mitochondrial Antiviral Signaling protein (MAVS) associated with long-term nonprogression in SIV-infected rhesus macaques

**DOI:** 10.1186/s12985-018-1069-5

**Published:** 2018-10-16

**Authors:** Miaomiao Zhang, Zhuotao Fu, Jiantao Chen, Boqiang Zhu, Ye Cheng, Linchun Fu

**Affiliations:** 1 College of Traditional Chinese medicine, Hebei University, Baoding, 071000 China; 2 Tropical Medicine Institute, Guangzhou University of Chinese medicine, Guangzhou, 510405 China; 3 The first Affiliated Hospital, Guangzhou University of Chinese medicine, Guangzhou, China

**Keywords:** Simian immunodeficiency virus, Disease progression, Activation, Inflammation, LTNP, MAVS

## Abstract

**Background:**

Abnormally increased immune activation is one of the main pathological features of acquired immunodeficiency syndrome (AIDS). This study aimed to determine whether long-term nonprogression (LTNP) suppresses the upregulation of immune activation and to elucidate the mechanisms whereby the LTNP state is maintained.

**Methods:**

For this study we selected 4 rhesus macaques(RMs) infected with simian immunodeficiency virus (SIV) that were long-term nonprogressors (LTNP); for comparison we chose 4 healthy RMs that were seronegative for SIV (hereafter referred to as the Control group), and 4 progressing infection (Progressive group) SIV RMs. We observed these animals for 6 months without intervention and explored the immunological and pathological differences among the 3 groups. A series of immune activation and inflammation markers—such as C- C chemokine receptor type 5 (CCR5), beta 2- microglobulin (β2-MG), Human Leukocyte Antigen - antigen D Related (HLA-DR), CD38, the levels of microbial translocation (LPS -binding protein), and MAVS—and histological features were monitored during this period.

**Results:**

Both SIV RNA and SIV DNA in the plasma and lymph nodes (LNs) of the LTNP group were at significantly lower levels than those of the Progressive group (*P* < 0.05). The CD4/CD8 ratio and CD4 cell count and proportion in the LTNP group were between those of the Progressive and Control groups (*P* < 0.05): that is, they were higher than in the Progressive group and lower than in the Control group. The LTNP macaques manifested slow progression and decreased immune activation and inflammation; they also had lower levels of CCR5, LPS-binding protein, and β2-MG than the Progressive RMs (*P* < 0.05). Activation of LTNP in both CD4+ and CD8+ T cells was significantly lower than in the Progressive group and closer to that in the Control group. The histological features of the LTNP macaques were also closer to those of the Control group, even though they had been infected with SIV 4 years earlier. These data point to low viral replication in the LTNP macaques but it is not static. The expression of MAVS in peripheral blood and LNs was lower in the LTNP group than that in the Progressive group (*P* < 0.01), and MAVS was positively correlated with SIV DNA in LNs (*P* < 0.05). This may reflect the low activation of T lymphocytes. It was speculated that MAVS may be the link between innate and acquired antiviral immunity in SIV infection.

**Conclusions:**

The LTNP RMs in our study were in a relatively stable state of low activation and inflammation, some biological progression with no disease events. This may have been associated with their low levels of the mitochondrial antiviral signaling protein (MAVS).

## Background

It has been found that some HIV-1-positive persons can go for more than 10 years without any progression of their disease, they maintain high stable CD4 cell counts and low viral loads in the absence of antiretroviral therapy. In which some patients are referred to as elite controllers (EC, < 50 copies/m L), another subset of non-progressing patients also maintain normal CD4+ T cell counts for several years, but in contrast to controllers these patients have on-going viral replication. This subset of patients is therefore termed Long Term Non-Progressors (LTNPs) [1, 2], which have long been the focus of research aiming to understand the natural mechanisms that can prevent progression of the disease.

LTNP has attracted a great deal of interest in hopes of identifying the mechanisms that contribute to the natural control of viral replication [3]. Various viewpoint regarding the progression of HIV disease in LTNP individuals have been proposed. Viral factors [4, 5], host genetics [6–8] and immune responses [9–11] have been associated with the control of HIV-1 replication and lack or slow disease progression. Research on LTNP may help clarify HIV/AIDS, which may lead to the development of immune therapy or a therapeutic vaccine in the future. MAVS (also called IPS-1 or VISA) is a key mediator of antiviral immunity following RIG-I and MDA-5 sensing of viral RNA [12, 13].MAVS signaling is known to play a role in control of number of viral infections, through the induction of type I interferons [14]. Despite the central role of MAVS in viral RNA-mediated interferon induction and innate and adaptive immune responses, MAVS was rarely studied in the context of HIV or SIV infection.

Our team has studied this phenomenon in a RM model of simian immunodeficiency virus (SIV) for more than 10 years. We found that some SIV-infected RMs (about 6–10% of these animals according to our experience) do not progress to simian acquired immunodeficiency syndrome (SAIDS), their viral loads decrease to undetectable levels, as is typical of LTNPs. But another group of SIV-infected macaques (also about 6–10% of these animals according to our experience) quickly progressed to SAIDS 3 to 6 months after being infected, they belong to the rapidly progressing (RP) group [15]. Most macaques survive for 1 to 3 years after infection, they belong to the progressing infection (Progressive) group. Once the set-point phase is reached following mucosal SIV challenge, the level of viral load predicts the rate of progression to AIDS [16–18]. We planned our research hoping that if we could determine the pathological mechanisms governing the 3 types of progression we might also be able to shed light on the principles and mechanisms of protective immunity, particularly with regard to the treatment of HIV/AIDS. The LTNP phenomenon may help to guide the design of immunotherapy, possibly leading to the development of new vaccines. In this study, we explored the immunological and pathological characteristics of untreated SIV-infected macaques with different patterns of disease progression, including Progressive and LTNP, in an effort to uncover the mystery of LTNP.

## Methods

### Animals

Colony-bred RMs (*Macaca mulatta*) of Chinese origin were housed at the nonhuman primate laboratory of Gaoyao experimental animal center (Guangdong, China). All animals were performed a full hematological analysis, and they had not occurred any disease within the past 2 months. They were subjected to complete physical examinations by the same experienced veterinarian and defined clinically healthy with no evidence of disease. All the animals included in the study were male, 3 to 6 years old, weighed 4 to 8 kg, and were seronegative for SIV, simian retrovirus (SRV), simian T-cell lymphotropic virus 1 (STLV-1), *Herpesvirus simiae* (B virus), and *Shigella* bacteria. X-ray examinations and skin tests (with purified protein derivative, or PPD) were performed at entry for all animals to exclude potential carriers of tuberculosis. Macaques were inoculated intravenously with 5 MID_100_ of SIVmac239 (the gift from Dr. Yongtang Zheng, Kunming Institute of Zoology, Chinese Academy of Sciences). The study was carried out in strict accordance with the recommendations in the guide for the care and use of laboratory animals of the National Institute of health. The protocol was approved by the institutional animal care and use committee, GaoYao Kangda laboratory animals science and technology co., ltd. (IACUC no: 113009)

### Blood samples and lymph nodes

Blood samples were collected from limb veins and placed in tubes containing 2 mmol/L EDTA-K3 anticoagulants and tubes without anticoagulants at 9:00- a.m. when the RMs were on an empty stomach. Some whole blood anti-coagulated with EDTA was used for flow cytometry analysis. The rest of the blood was separated and preserved in plasma and serum in a − 80 °C freezer for further analysis. Under anesthesia (10 mg/kg ketamine and Sumianxin 0.05–0.1 mg/kg), a whole lymph node was collected in sterile conditions from axillary or inguinal. Biopsy specimens were divided into 3 parts, the first being immediately fixed in 4% neutral buffered paraformaldehyde, followed by paraffin embedding and slicing routinely. The second part was frozen in liquid nitrogen in preparation for checking by Western blot, and the third part was used to check T-cell subsets by flow cytometry. Euthanasia protocol: Animals were anesthetized with ketamine (10 mg/kg) and Sumianxin (0.05–0.1 mg/kg), then killed via intravenous injection of sodium pentobarbital (25–45 mg/kg) and femoral artery bloodletting

### Viral loads

Cell-associated SIV DNA was quantified by a quantitative PCR. The plasma virus RNA was extracted with RNAout kit(TIANDZ Inc., Mianyang city of Sichuan Province, China),which mainly contained guanidinium isothiocyanate and LiC1.Plasma SIV RNA was quantified by a quantitative RT-PCR assay as descried previously [19],with primers (sense, 5′- GGAGGAAATTACCCAGTACAACAAAT -3′; antisense,5’-CCTGAAATCCTGGCACTACTTCT-3′) and probe (5’-FAM-ACTATGTCCACCTGCCATTAAGCCCGAGA-TAMRA-3′)for SIVmac239 and primers (sense,5’-GAAATCCCATCACCATCTTCCAGG-3′; antisense, 5′- GAGCCCCAGCCTTCTCCATG -3′) for GAPDH [20] using a 7500 fast real-time PCR system (applied biosystems, Foster City, CA), TIANamp genomic DNA kit (cat:DP304–03, lot:M2016) and 204,054-QuantiFast SYBR green PCR kit(400)(QIAGEN)for cell-associated SIV DNA, RevertAidTM reverse transcriptase and maxima probe/ROX q PCR master mix(2×)(Fermentas)for plasma SIV RNA. The sensitivity of the plasma viral RNA detection utilizing this technique was determined to be 50 copies /m L plasma

### Flow Cytometry

Flow cytometry analysis was carried out with FACScalibur (Becton Dickinson, San Jose, CA) using the following fluorescence-labeled monoclonal antibodies: CD3-APC (clone SP34–2), CD4-Percp (clone L200), and CD8-PE (clone RPA-T8). These were purchased from BD biosciences, HLA-DR-PEcy7 (L243, biolegend),CD38-FITC (AT-1, stemcell). CellQuest pro software was used for the analysis of the flow cytometric data

### Plasma LPS-binding protein levels

Plasma LPS-binding protein (LBP) was quantified with a commercially available ELISA kit (Biometec, Greifswald, Germany) according to the manufacturer’s protocol. We ran samples in duplicate and zeroed the reader with blank wells using the MK3 Thermo Scientific microplate reader (thermo fisher scientific, Waltham, MA, US). The absorbance was read at 450 nm (OD450). The linear regression equation of the standard concentration was the horizontal coordinate, and the OD value was the vertical coordinate. Then the concentrations of samples were calculated according to the standard curve

### Serum β2-MG levels

Beta-2 microglobulin (β2-MG) levels in serum were measured by radioimmunoassay in 3 groups. The sample, marker, and antibody were added to the test tube. Then the separation agent was added after the reaction was balanced. After centrifugation, the radioactive intensity in the precipitate was measured by a scintillation counter (SN-695B γ)

### Western blot

The protein was extracted from peripheral serum using a serum protein extraction kit (BB-3138-2, BestBio), and the protein was extracted from lymph nodes using a the total protein extraction kit (SD-001, invent biotechnologies). The expression of CCR5 (ab1673, Abcam) and MAVS (ab31334, Abcam) in peripheral blood and LNs was detected by Western blot. This was achieved with a rabbit α-syn polyclonal antibody diluted at 1:1000 and then with the appropriate peroxidase-conjugated secondary antibodies diluted at 1:2000. Normalization was made against glyceraldehyde 3-phosphate dehydrogenase (GAPDH) expression diluted at 1:1000 (Santa Cruz biotechnology, Dallas, TX). The developed films were scanned as tiff images in 8-bit gray-scale format at a setting of 300 dpi and the band intensities were measured by image J software (National Institutes of Health, Bethesda, MD)

### Histopathological examination

The lymph node specimens were embedded in paraffin and processed using hematoxylin and eosin (HE) staining. They were then examined under a light microscope (Leica, Wetzlar, Germany)

### Statistical analysis

Statistical analysis was performed using SPSS 17.0, and the data was expressed as mean ± SD. The analysis of variance(ANOVA)for repeated measures was used for comparisons among the means of indexes. *P* < 0.05 was considered statistically significant

## Results

### Plasma viral loads and CD4 cell counts in the LTNP group

**Fig. 1:**
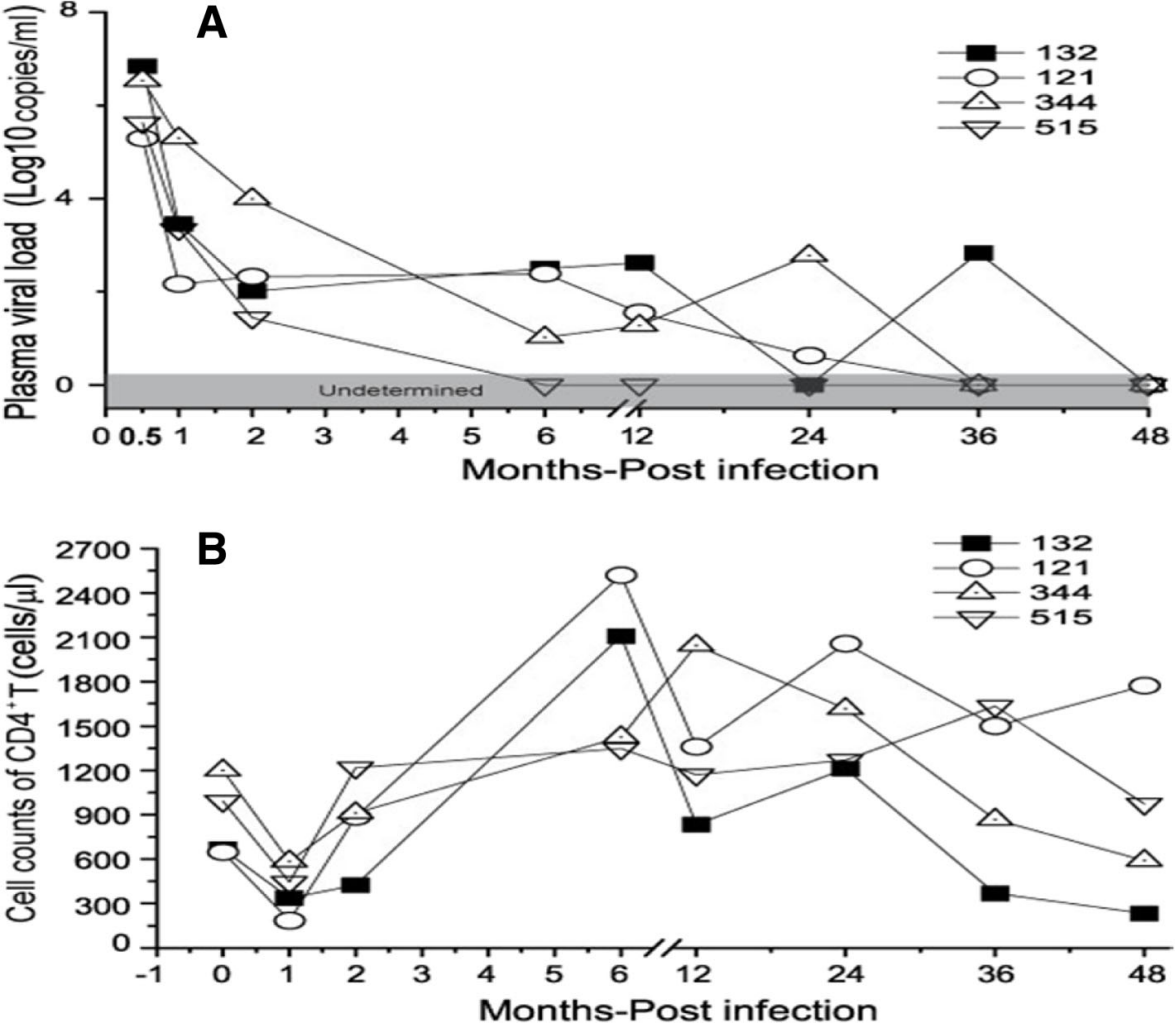
Plasma viral loads (**a**) and CD4 cell counts (**b**) of each animal after SIV infection in LTNP group

For our study, we selected 4 LTNP RMs that had been infected with SIVmac239 virus 4 years earlier. None of them had diarrhea, emaciation, changes in appetite or food intake, fatigue, fear of cold, edema, or alopecia, which was a series of possible symptoms of SIV infected RMs. They did not receive antiretroviral medication and survived with low levels of SIV RNA in the plasma and had stable CD4+ T-cell counts. They manifested no SIV-related illness (Fig. 1).

### Slow progression in the LTNP group

In investigating the reasons for LTNP, we explored the immunological and pathological differences among LTNP, Control, and Progressive RMs, and the study started 3 months after SIV infection with the Progressors. According to convention, we tested their viral loads and CD4 cell counts to evaluate the characteristics of LTNP. We found that both SIV RNA and SIV DNA in plasma and the LNs of the LTNP group were significantly lower than those of the Progressive group. There was no detectable plasma viral load in the LTNP group. However, SIV DNA was detectable in the LNs; The analysis of variance(ANOVA)for Repeated Measures showed that it was significantly lower than that of the Progressive group (*P* < 0.05) (Table 1).

No. 579 rhesus of the Progressive group was dead on the twenty-fifth day after the observation ending. At autopsy, it was found that this animal had severely gastrointestinal flatulence; there was a 1- × 1-cm perforation on the right side of the stomach. The Control group and the LTNP group were alive and healthy.

**Fig. 2:**
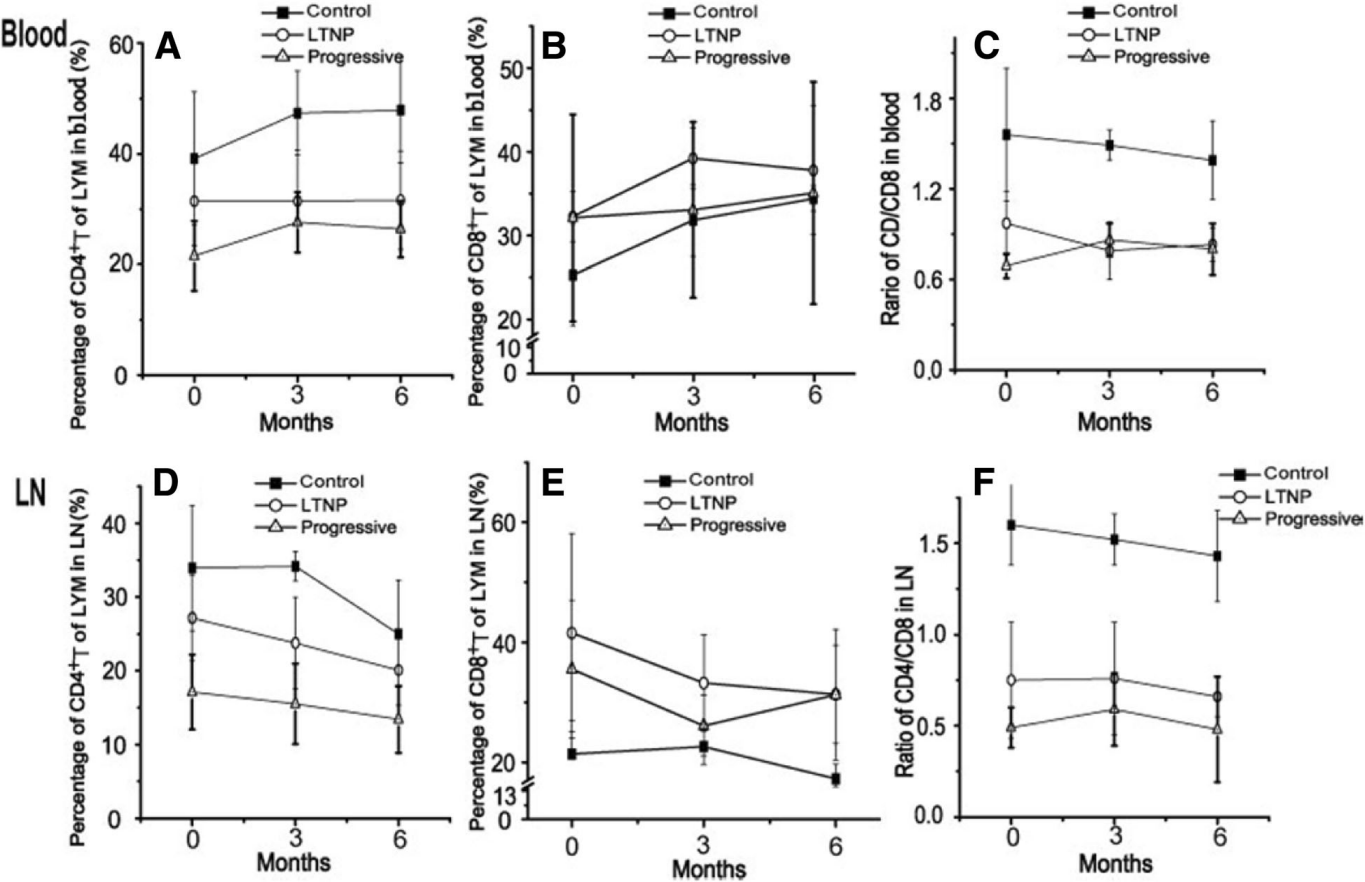
T-cell subsets in peripheral blood and LNs in different groups The percentages of T cells in peripheral blood (**a**) CD4+T of LYM, (**b**) CD8+T of LYM, (**c**) CD4/CD8 ratios. The percentages of T cells in LNs (**d**) CD4+T of LYM, (**e**) CD8+T of LYM, (**f**) CD4/CD8 ratios

The CD4/CD8 ratios and CD4 counts in the LTNP group were in line with our expectations: they were between those of the Progressive and Control groups. The percentages of T cells (CD4 and CD4/CD8 ratios) in peripheral blood and LNs from the LTNP group were between those of the Control and Progressive groups (*P* < 0.05); The numbers of the Progressive group were significantly lower than those of the LTNP group, and the numbers of both groups were lower than those of the Control group (Fig. 2, Tables 2 and 3).

**Table 1:** Plasma and LN Viral Loads in different groups

Group	Study initiation (months)	Plasma viral loads (Log_10_RNA copies/mL)	LN (Log_10_DNA /10^6^ cell)
LTNP (*n* = 4)	0	Undetermined	2.39 ± 0.73
	3	Undetermined	2.91 ± 0.65
	6	Undetermined	2.77 ± 0.52
Progressors (*n* = 4)	0	4. 40±0.79	3.78 ± 0.39
	3	4.70 ± 0.13	3.90 ± 0.31
	6	4.60 ± 0.68	3.80 ± 0.37

The 0 point of Progressors occurred 3 months after SIV infection

Between-Subjects Effects in LN, F= 11.800, *P* = 0.014 < 0.05 in the Analysis of variance (ANOVA) for Repeated Measures

**Table 2:** Comparison of percentages of peripheral blood T-cell subsets among three groups

	Group	Study initiation(months)			Between-Subjects Effects	
		0	3	6	F	*P*
CD4%	Control (*n* = 4)	39.14 ± 12.06	47.35 ± 7.59	47.93 ± 9.56	5.739	0.028*
	LTNP (*n* = 4)	31.41 ± 8.04	31.46 ± 9.23	31.60 ± 8.89		
	Progressors (*n* = 4)	21.54 ± 6.31	27.61 ± 5.45	26.40 ± 5.10		
CD8%	Control (*n* = 4)	25.29 ± 6.07	31.82 ± 4.27	34.41 ± 1.52	0.486	0.632
	LTNP (*n* = 4)	32.27 ± 3.03	39.24 ± 3.65	37.79 ± 7.70		
	Progressors (*n* = 4)	32.12 ± 12.36	33.07 ± 10.50	35.08 ± 13.27		
CD4/CD8	Control (*n* = 4)	1.56±0.44	1.49 ± 0.10	1.39 ± 0.26	21.093	0.001**
	LTNP (*n* = 4)	0.97 ± 0.21	0.79 ± 0. 19	0.83 ± 0.11		
	Progressors (*n* = 4)	0.69 ± 0.08	0.86 ± 0. 11	0.80 ± 0.17		

*,*P* < 0.05 in the Analysis of variance (ANOVA) for Repeated Measures

**,*P* < 0.01 in the Analysis of variance (ANOVA) for Repeated Measures

**Table 3:** Comparison of percentages of peripheral LN T-cell subsets among three groups

	Group	Study initiation(months)			Between-Subjects Effects	
		0	3	6	F	*P*
CD4%	Control (*n* = 4)	33.94 ± 8.49	34.16 ± 1.99	25.02 ± 7.28	12.252	0.004**
	LTNP (*n* = 4)	27.17 ± 5.79	23.77 ± 6.20	20.09 ± 4.78		
	Progressors (*n* = 4)	17.12 ± 5.09	15.52 ± 5.46	13.41 ± 4.55		
CD8%	Control (*n* = 4)	21.37 ± 5.60	22.61 ± 3.01	17.25±2.47	3.453	0.083
	LTNP (*n* = 4)	41.62 ± 16.47	33.24 ± 8.03	31.35 ± 8.20		
	Progressors (*n* = 4)	35.54 ± 11.45	26.10 ± 5.05	31.26 ± 10.91		
CD4/CD8	Control (*n* = 4)	1.6 ± 0.22	1.52 ± 0.14	1. 43±0.25	40.946	0.000**
	LTNP (*n* = 4)	0.75 ± 0.32	0.76 ± 0.31	0.66 ± 0.11		
	Progressors (*n* = 4)	0.49 ± 0.11	0.59 ± 0.20	0. 48 ± 0.29		

**,*P* < 0.01 in the Analysis of variance (ANOVA) for Repeated Measures

### Lower immune activation and inflammation in the LTNP group than in progressors

**Fig. 3:**
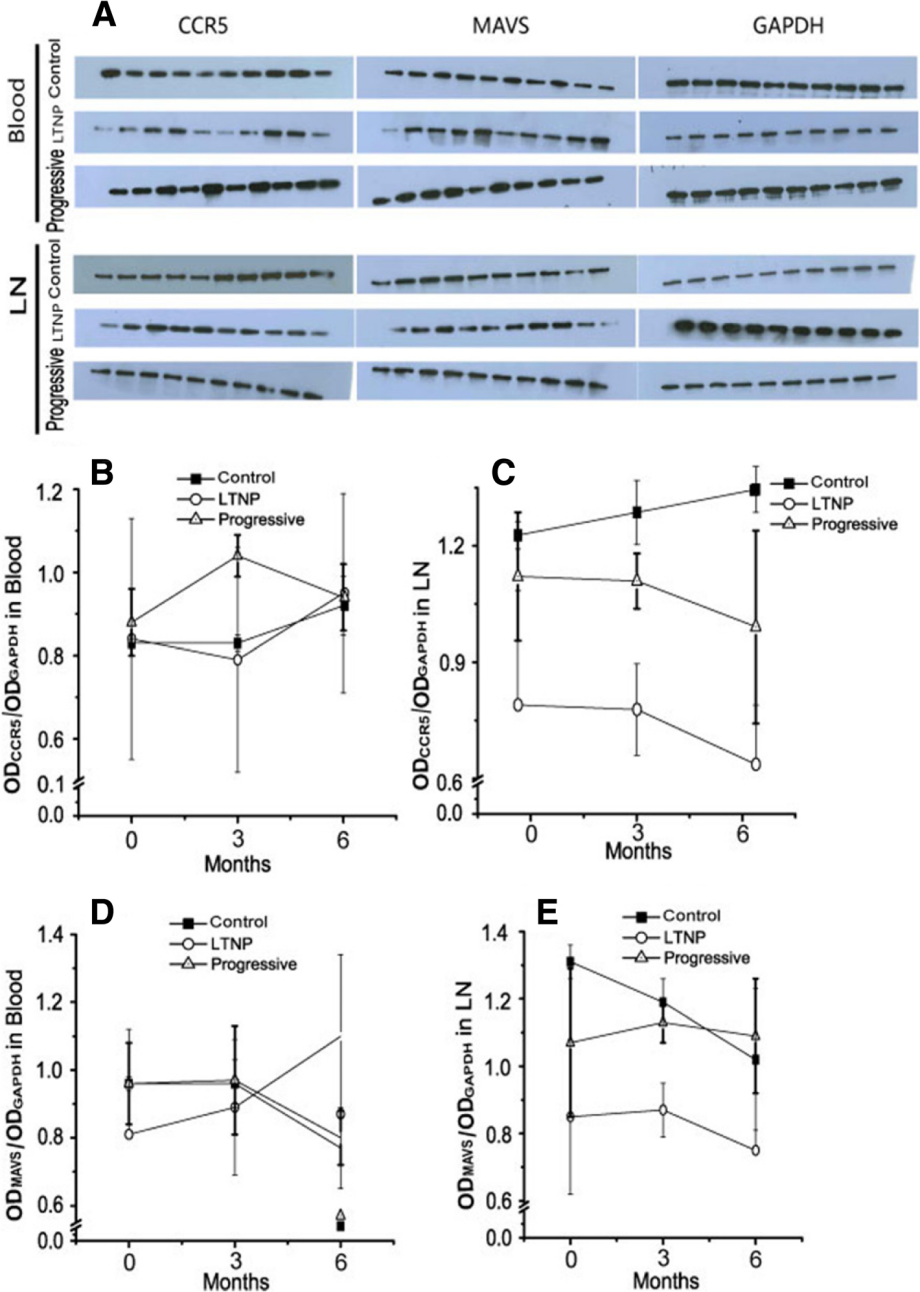
CCR5 and MAVS in peripheral blood and LNs in different groups (**a**) The bands in place on the gels of CCR5 and MAVS in peripheral blood and LNs. The expression of CCR5 in the peripheral blood (**b**) and LNs (**c**). The expression of MAVS in the peripheral blood (**d**) and LNs (**e**)

The bands in place on the gels of CCR5 and MAVS in peripheral blood and LNs in the different groups were showed (Fig. 3a). The expression of CCR5 in the peripheral blood among three groups there was no significant difference (Fig. 3b), however, the levels of CCR5 in LN were quite different (LTNP < Progressor < Control) (Fig. 3c) (Table 4). The expression of CCR5 in LNs was lower in the LTNP than in the Progressive group (*P* < 0.01), suggesting that this may be an important feature of LTNP and may even participate in the formation of LTNP. The mitochondrial antiviral signaling protein (MAVS), also known as IPS-1/VISA/CARDIF, being a common intermediary signaling protein [21], in our study, similar to CCR5, the expression of MAVS in the peripheral blood among three groups there was no significant difference (Fig. 3d), but MAVS in LN was significantly lower in the LTNP than in the Progressive group and Control group (*P* < 0.01) (Fig. 3e) (Table 5), possibly resulting in lower inflammatory cytokines in LTNP LNs, thereby reducing lymphocyte activation and disease progression.

**Fig. 4:**
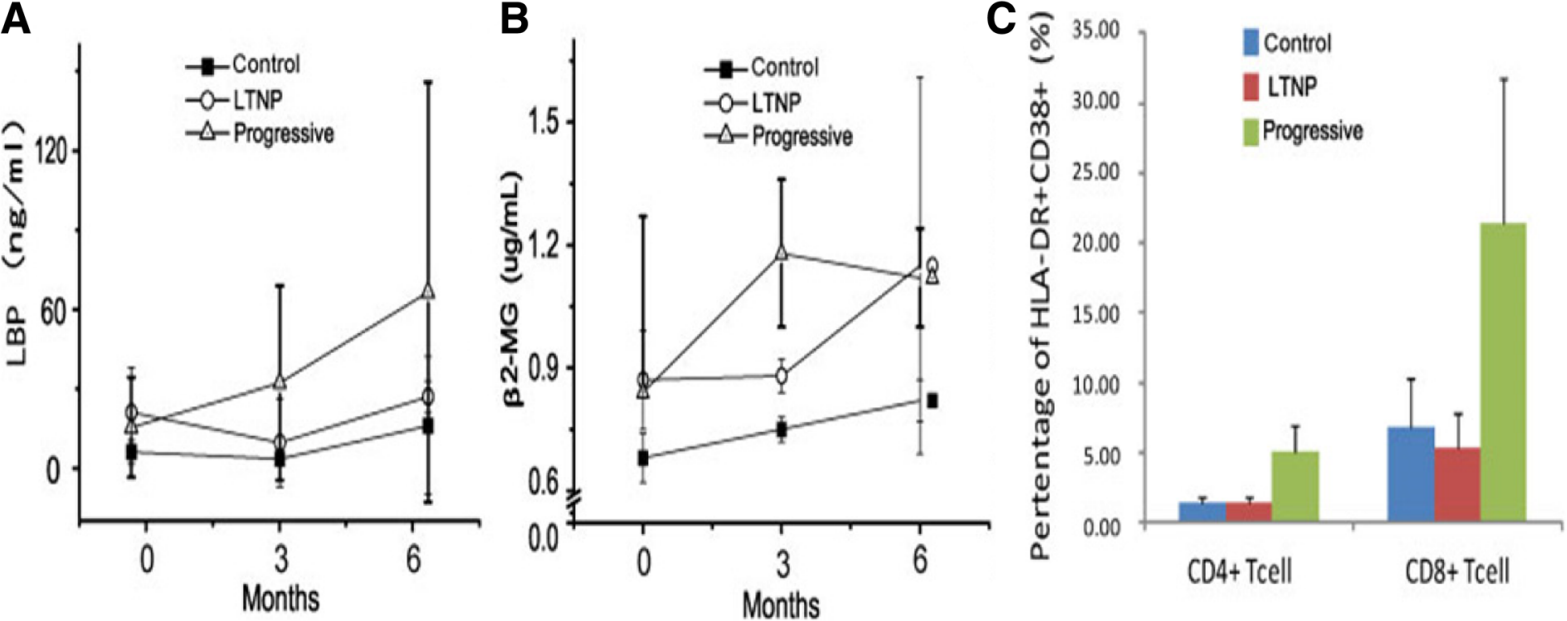
Activation and inflammation indexes in different groups (**a**) levels of LBP in different groups, (**b**) levels of β2-MG in different groups, (**c**) percentages of HLA-DR and CD38 in different groups

In order to verify the difference in activation and inflammation in each group, we detected a series of relevant indexes, including LPS, β2-MG, HLA-DR, and CD38. LPS is related to the natural immune activation of HIV, it represents the level of immune activation and bacterial migration. Damage to the intestinal mucosa results in the translocation of microbes from the intestinal lumen into the circulation [22]. To measure microbial translocation, we quantified the concentration of LBP throughout infection in the plasma by enzyme-linked immunosorbent assay (ELISA). LBP is produced in response to bacterial LPS and thus indicates the presence of microbial products in the bloodstream [23], and LBP is a relatively reliable measurement of systemic microbial translocation in the plasma of nonhuman primates [24]. Our results showed that LTNP RMs had lower levels of LBP compared with Progressive RMs, but higher levels than the Control macaques (*P* < 0.05) (Fig. 4a, Table 6). β2-MG is a kind of immune function index, and its higher level represents stronger immune activation [25, 26]. Our study was consistent with previous research: we showed that the level of β2-MG in the LTNP group was between that of the Control group and the Progressive group (higher than Control, lower than Progressive) (*P* < 0.05) (Fig. 4b, Table 6). Then we further investigated the activation of T lymphocytes at the 6-month time point. HLA-DR and CD38 are marker molecules for T-cell activation and, in accordance with our expectations, we found that the activation of LTNP both in CD4+ and CD8+ T cells was significantly lower than in the Progressive group and closer to that in the Control group (Fig. 4c).

**Table 4:** Comparison of CCR5 in peripheral blood and LN among three groups (OD_CCR5_/OD_GAPDH_)

Tissue	Group	Study initiation(months)			Between-Subjects Effects	
		0	3	6	F	*P*
Blood	Control (*n* = 4)	0.83 ± 0.01	0.83 ± 0.02	0.92 ± 0.07	2.051	0.191
	LTNP (*n* = 4)	0.84 ± 0.29	0.79 ± 0.27	0.95 ± 0.24		
	Progressors (*n* = 4)	0.88 ± 0.08	1.04 ± 0.05	0.94 ± 0.08		
LN	Control (*n* = 4)	1.18 ± 0.03	1.23 ± 0.07	1.28 ± 0.05	52.114	0.000**
	LTNP (*n* = 4)	0.81 ± 0.25	0.80 ± 0.10	0.68 ± 0.13		
	Progressors (*n* = 4)	1.09 ± 0.14	1.08 ± 0.06	0.98 ± 0.21		

**, *P* < 0.01 in the Analysis of variance (ANOVA) for Repeated Measures

**Table 5:** Comparison of MAVS in peripheral blood and LN among three groups (OD_MAVS_/OD_GAPDH_)

Tissue	Group	Study initiation(months)			Between-Subjects Effects	
		0	3	6	F	*P*
Blood	Control (*n* = 4)	0.96 ± 0.02	0.96 ± 0.07	0.77 ± 0.12	0.258	0.779
	LTNP (*n* = 4)	0.81 ± 0.31	0.89 ± 0.20	1.10 ± 0.24		
	Progressors (*n* = 4)	0.96 ± 0.12	0.97 ± 0.16	0.80 ± 0.08		
LN	Control (*n* = 4)	1.31 ± 0.05	1. 19±0.07	1.02 ± 0.21	4.132	0.002**
	LTNP (*n* = 4)	0.85 ± 0.23	0.87 ± 0.08	0.75 ± 0.26		
	Progressors (*n* = 4)	1.07 ± 0.22	1.13 ± 0.06	1.09 ± 0.17		

**,*P* < 0.01 in the Analysis of variance (ANOVA) for Repeated Measures

**Table 6:** Comparison of activation and inflammation indexes in peripheral blood among three groups

	Group	Study initiation(months)			Between-Subjects Effects	
		0	3	6	F	*P*
LBP (ng/mL)	Control (*n* = 4)	9.24 ± 4.27	6.79 ± 1.76	18.70 ± 24.72	0.968	0.420
	LTNP (*n* = 4)	23.50 ± 16.10	12.58 ± 15.85	29.08 ± 5.55		
	Progressors (*n* = 4)	18.13 ± 17.89	34.05 ± 34.84	66.60 ± 75.41		
β2-MG (ug/mL)	Control (*n* = 4)	0.68 ± 0.06	0.75 ± 0.03	0.82 ± 0.05	5.946	0.026*
	LTNP (*n* = 4)	0.87 ± 0.12	0.88 ± 0.04	1.15 ± 0.46		
	Progressors (*n* = 4)	0.84 ± 0.43	1.18 ± 0.18	1.12 ± 0.12		

*,*P* < 0.05 in the Analysis of variance (ANOVA) for Repeated Measures

### Histological features of macaques in the different groups

**Fig. 5:**
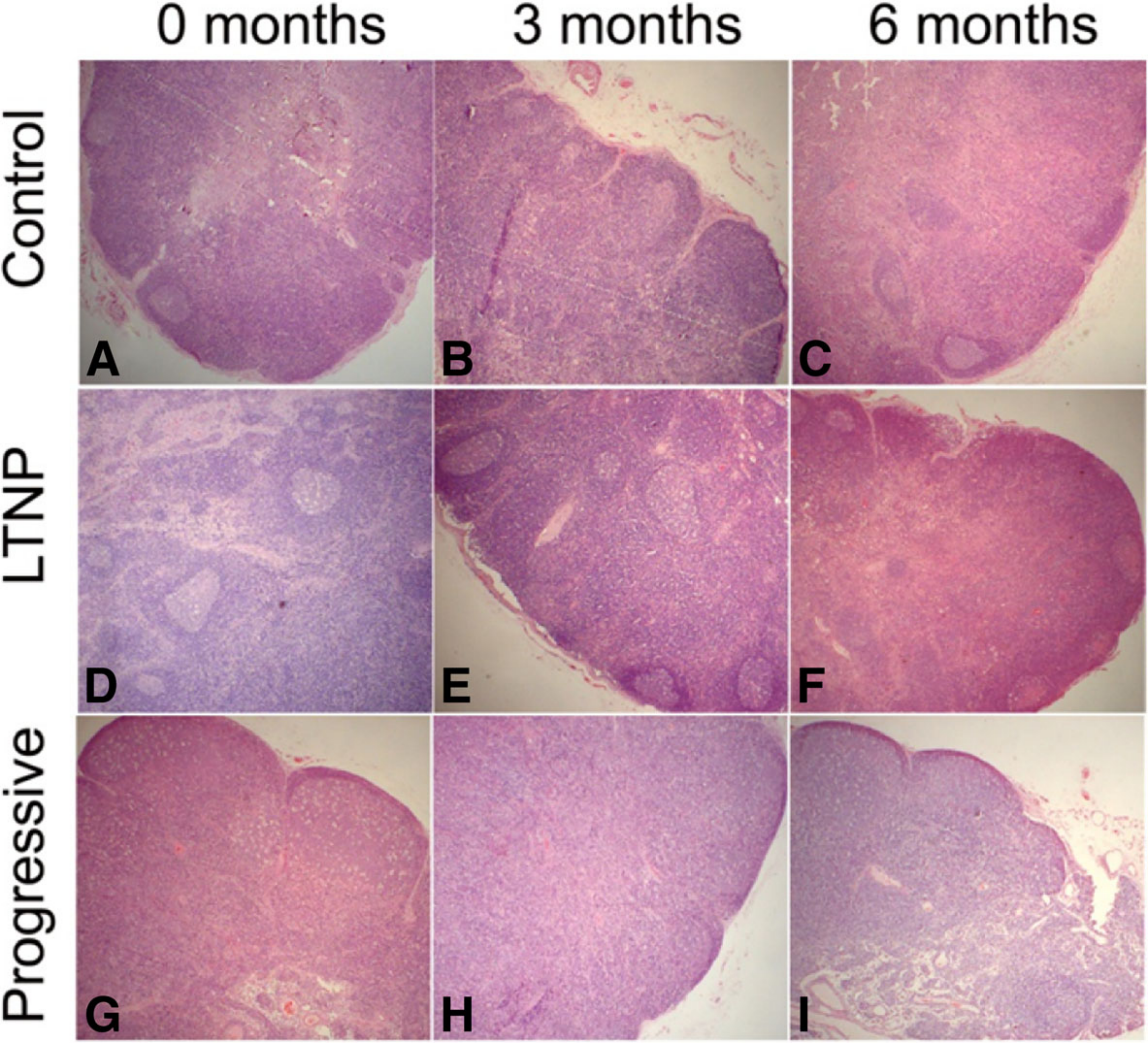
Histopathology of lymph nodes in different groups (H&E staining, × 40 magnification) The histological features of lymph 0 months (**a**), 3 months (**b**), 6 months (**c**) in Control group; The histological features of lymph 0 months (**d** ), 3 months (**e**), 6 months (**f**) in LTNP group; The histological features of lymph 0 months (**g**), 3 months (**h**), 6 month (**i**) in Progressive group

The histological features of lymph follicles and germinal centers (GC) in LNs were examined at 0, 3 and 6 months. Lymph follicles and GC in LNs were well-preserved in Control macaques (Fig. 5a-c). Lymph follicle and GC were relatively well-preserved in LTNP macaques (Fig. 5d-f). However, lymph follicles were destroyed and GC disappeared in Progressive RMs (Fig. 5g-i).

The histological features of LTNP RMs were closer to those of the Control group even though they had been infected with SIV 4 years earlier. Presenting features of the LNs included a small size, thin cortex, and lymphoid follicles; the germinal centers were also small. The lymph nodes of the Progressive RMs, by comparison, were large, with cortical thickening, enlarged lymphoid follicles, and larger germinal centers. This result reflected the lower state of immune activation in the LTNP macaques. These may be the fundamental features of of long-term nonprogression.

## Discussion

Previous research [27, 28] has shown that excessive immune activation increased the susceptibility of T cells to HIV, supporting viral replication and accelerating the progression to AIDS. Excessive immune activation provides more susceptible cells, and the products of immune activation (i.e., inflammatory cytokines and apoptotic molecules) can lead to strong bystander immune pathological injury. The activation of induced apoptosis mechanisms can also lead to large losses of lymphocytes, eventually causing immune system dysfunction. The immune response is a double-edged sword that can not only protect the body but also hurt it. Excessive immune activation that is not the result of the immune system homeostasis is harmful to the host. Therefore the further study of whether LTNP suppresses the upregulation of activation could be an important step for discovering new therapeutics. In our study, we monitored a series of immune activation markers—such as CCR5, β2-MG, HLA-DR, and CD38. CCR5 is a surface protein located on the plasma membrane of white blood cells that regulates T-cell and monocyte/macrophage cell migration, proliferation, and immune function. It is mainly expressed on activated memory T-lymphocytes, monocytes, and immature dendritic cells in the cell membrane. CCR5 is a co-receptor for early HIV-1 infection, which is closely related to inflammation. It plays an important role in determining the migration of T-cells and susceptibility to HIV infection and disease progression [29, 30]. Levels of CCR5 on cell surface determines bystander apoptosis of cells via HIV Env, with higher CCR5 expression associated with increased bystander apoptosis in vitro[31]. Increased cell surface CCR5 levels would likely support higher virus replication [32], and levels of CCR5 can affect HIV-mediated CD4 loss [31, 33].CCR5 promoter activity correlates with HIV disease progression by regulating CCR5 cell surface expression and CD4 T cell apoptosis [34]. Interestingly, the HIV-exposed but uninfected subjects (ESNs) had significantly lower frequencies of CCR5+ CD4+ and CD8+ T cells than unexposed individuals [35], and antibodies to CCR5 have been detected in (ESNs) [36]. Furthermore, a role of anti CCR5 antibodies in mediating CCR5 downregulation has been demonstrated in some LTNPs: researchers found that there were anti-CCR5 antibodies in 23.5% of the LTNPs, but not in the other populations (including HIV progressors and HIV-negative controllers) studied (*P* < 0.001). Follow-up studies showed that the loss of anti-CCR5 antibodies occurred in some subjects, and this loss was significantly associated with a progression toward disease, whereas subjects who retained anti-CCR5 Abs maintained their LTNP status [37], further supporting a role for CCR5 levels in disease progression. In our study, we observed that the expression of CCR5 was lower in the LTNP than in the Progressive group, suggesting that this may be an important feature of LTNP and may even participate in the formation of LTNP. While Control RMs having higher CCR5 in their lymph nodes, which suggest that CCR5 was down regulated in infected macaques and this down regulation maybe more important in LTNP than in Progressors.

Due to the lack of data of CCR5 expression in LTNP RMs pre-infection, it was hard to determine whether the lower CCR5 expression in LTNP RMs contributed to the protection against SIV progression, or it was only the conequence of lower SIV load and lower immune activation in LTNP animals compared to Progressors. Maybe it was just virus preferentially destroyed CCR5 cells. Since we had studied the expression of CCR5 only by Western Blot, So we cannot differentiate between the different CCR5 expressing T cell subsets which perhaps may explain some of the discrepancies. Further research is in need to answer the question. The concentration of β2-MG is quite stable in normal human blood [38], but it is significantly higher in HIV/AIDS patients than in normal people [39]. Furthermore, the development of HIV is associated with a higher β2-MG level [40]. With HIV progression, the activation of lymphocytes increases along with the β2-MG level, and the mortality rate is also increased [25, 26]. Our study was consistent with previous research: we showed that the level of β2-MG in the LTNP group was between that of the Control group and the Progressive group. We noted CCR5, β2-MG, HLA-DR, and CD38 lower level expression in LTNP than that in Progressive group, which could also be a consequence of the suppressed viral replication in LTNP. These immune response were responsible, at least in part, for the maintenance of intact lymphoid tissue architecture and low levels of viral burden.

Chronic immune activation is a characteristic feature of progressive HIV disease. It has been well recognized that injury to the immune component of the gastrointestinal mucosal surface, along with damage to the intestinal epithelial microenvironment with its antimicrobial functions, may affect systemic immune activation during the chronic phase of HIV infection through the increased translocation of luminal microbial products [41]. Within the first few weeks of infection, the bulk of CD4 T-cell depletion occurs rapidly and is predominantly localized to the gastrointestinal tract [42, 43]. Notably, the extent of mucosal CD4 T-cell depletion in pathogenic SIV infection of rhesus macaques determines the rate of progression to AIDS [44]. Thus, there is an early breach in the integrity of the mucosal immune system. Lipopolysaccharide (LPS), a major component of Gram-negative bacterial cell walls and a potent immunostimulatory product [45], can be quantitatively assessed in plasma and commonly measured to determine the degree of microbial translocation. Circulating lipopolysaccharide is significantly increased in chronically HIV-infected individuals and in simian immunodeficiency virus (SIV)-infected rhesus macaques, and the increased lipopolysaccharide is bioactive in vivo and correlates with measures of innate and adaptive immune activation [46].In chronic HIV infection, a poorly controlled translocation of bacterial products, as measured by LPS binding protein, occurs and correlates with immune activation markers, which in turn correlate with disease progression [47]. Plasma LPS levels correlate with systemic immune activation, which drives chronic HIV infection [28]. The results of our study were consistent with these findings. The levels of microbial translocation, as measured by LPS-binding protein in our LTNP group were between those of the Control and Progressive groups (higher than Control, lower than Progressive). Consistent with these reports, we also observed a markedly lower level of LBP in LTNP animals than in progressive infection animals, although the levels remained higher than those of control RMs. These data suggest that viral replication is low in LTNP macaques but not static. Previous studies had shown that high levels of plasma LPS alone cannot induce T-cell activation. HIV/SIV disease seems to be marked by constant bacterial translocation and immune activation. Therefore it is inflammation that causes LPS to rise in SIV RMs; LPS to rise is the result, not the cause [48].

This leads to the question of what causes the conditions previously described. Our team speculated MAVS was involved in driving low immune activation in LTNP. MAVS mediates activation of nuclear factor kappa B (NF-κB) and interferon regulatory factors (IRFs) and the induction of interferons in response to viral infection [49]. This process eventually leads to the activation of various antiviral genes, thus inhibiting viral replication and transmission. The innate immune system recognizes nucleic acids (SIV RNA in peripheral blood and LNs) during SIV infection and stimulates cellular antiviral responses, while RIG-1–like receptors (RLRs) bind viral RNA and initiate the antiviral immune response through their interaction with MAVS. MAVS activates restriction factors which could limit viral replication, and that HIV and probably SIV have developed strategies to evade MAVS. Researchers from the Netherlands have found that HIV-1 can evade the host’s antiviral immune response by blocking the MAVS signaling pathway, and thereby accelerate HIV-1 replication in infected individuals [50]. Previous study by Gupta S et al. Evaluated a constitutively active form of MAVS, and examined the ability of this construct to induce innate immune responses that inhibit HIV-1 viral replication, and that inhibiting HIV-1 replication via type I Interferon secretion and induction of HIV-1 restriction factors [51]. Solis M et al. Have demonstrated a novel protease-dependent mechanism employed by HIV-1 to counteract the early IFN response to viral RNA in infected cells, by virtue of removing RIG-I from the cytosol, protease may impede RIG-I interaction with the mitochondrial adaptor MAVS and thus disconnect early innate antiviral signaling [52]. Furthermore, apoptosis of infected cells via MAVS has also been postulated as an antiviral defense. On the other hand, activation of MAVS has been implicated in some autoimmune disorders. Excessive activation of MAVS-mediated antiviral signaling leads to dysfunction of mitochondria as well as cell apoptosis [53], which likely lead to the pathogenesis of autoimmunity. MAVS activates NF-κB pathway and induces immune activation and inflammation [54]. Therefore the body must maintain the balance of its natural antiviral immune responses [55]. MAVS monitors intestinal commensal bacteria and induces an immune response that plays a dominant role in maintaining tissue homeostasis and protection against colitis [56]. Many viruses circumvent the innate antiviral response by using viral proteins to antagonize the RIG-I/MAVS pathway at different levels [57]. Viral proteases, in particular, are known to abrogate the RIG-I/MAVS pathway through the direct degradation of the cleavage of the adaptor MAVS [58, 59]. The hepatitis C virus NS3-4A protease complex specifically targets MAVS/IPS-1/VISA/Cardif for cleavage as part of its immune evasion strategy, which results in its dissociation from the mitochondrial membrane and disrupts signaling to the antiviral immune response [58]. The PCBP2-AIP4 axis defines a new signaling cascade for MAVS degradation and ‘fine tuning’ of antiviral innate immunity [60]. In our study, there were higher MAVS in control macaques than that in LTNP and Progressors animals, particularly in lymph nodes, but lower activation markers than the LTNP and Progressors animals. This suggest that MAVS are down regulated in infected macaques and this down regulation maybe more important in LTNP than in Progressors. Due to the lack of data of pre-infection MAVS levels in LTNP RMs, it is hard to determine whether higher MAVS expression in Control RMs compared to LTNP and Progressor was caused by SIV infection or it’s only the congenital characteristics of LTNP RMs, it merits further research in the future. MAVS regulation is essential for the prevention of excessive harmful immune responses. In our study, MAVS was significantly lower in the LTNP group, which may be correlated with the low activation of T lymphocytes. Possibly because our sample size was relatively small, there was no statistical difference between MAVS and some activation indexes. However, it was still a useful exploration. It may be worthwhile to increase the sample size in future studies. There was not an exact explanation for why the MAVS level was higher at 6 month in blood in the LTNP than the Control and Progressive groups. The reason was not clear. Actually, our team is researching on it, but we have not found the answer yet. We speculate that this may be correlated with the regulation of the transcriptional level.

Our study provides novel insights on MAVS, which may be the link between Innate and acquired antiviral immunity in SIV infection. However, the mechanism of MAVS regulation at the mitochondria remains unknown. The effect of the preinfection expression of MAVS on postinfection disease progression is also unknown. In the future, it will be necessary to study the upstream and downstream molecule of MAVS and explore the mechanisms that control MAVS.

## Conclusions

This study aimed to determine whether LTNP suppress the upregulation of immune activation and to elucidate the mechanisms whereby the LTNP state is maintained. We found the LTNP were in a relatively stable state of low activation and inflammation, some biological progression with no disease events. The expression of MAVS in peripheral blood and LNs was lower in the LTNP group than that in the Progressive group. This may reflect the low activation of T lymphocytes. MAVS was speculated to be involved in driving low immune activation in LTNP. Our study provides novel insights on MAVS, which may be the link between Innate and acquired antiviral immunity in SIV infection.

## Abbreviations

AIDS: Acquired immunodeficiency syndrome
B virus: *Herpesvirus simiae*
HLA-DR: Human Leukocyte Antigen - antigen D Related
IRF3/7: Interferon regulatory factors 3 and 7
IRFs: Interferon regulatory factors
LNs: Lymph nodes
LPS: Serum endotoxin
LTNP: Long-term nonprogression
MAVS: Mitochondrial antiviral signaling protein
NF-Κb: Nuclear factor kappa B
PRRs: Pattern-recognition receptors
RIG-I: Retinoic acid–inducible gene-I
RMs: Rhesus macaques
RP: Rapidly progressing
SAIDS: Simian acquired immunodeficiency syndrome
SIV: Simian immunodeficiency virus
SRV: Simian retrovirus
STLV-1: Simian T-cell lymphotropic virus 1
TLRs: Toll-like receptors
β2-MG: Beta 2- microglobulin
